# Second Generation I-Body AD-214 Attenuates Unilateral Ureteral Obstruction (UUO)-Induced Kidney Fibrosis Through Inhibiting Leukocyte Infiltration and Macrophage Migration

**DOI:** 10.3390/ijms252313127

**Published:** 2024-12-06

**Authors:** Qinghua Cao, Michael Foley, Anthony J. Gill, Angela Chou, Xin-Ming Chen, Carol A. Pollock

**Affiliations:** 1 Renal Medicine, Kolling Institute of Medical Research, Sydney Medical School, Faculty of Medicine and Health, University of Sydney, Royal North Shore Hospital, St Leonards, NSW 2065, Australia; qinghua.cao@sydney.edu.au (Q.C.); xin-ming.chen@sydney.edu.au (X.-M.C.); 2 The Department of Biochemistry and Genetics, La Trobe Institute for Molecular Science, La Trobe University, Melbourne, VIC 3086, Australia; m.foley@latrobe.edu.au; 3 AdAlta Limited, LIMS2 Building, Science Drive, La Trobe University, Melbourne, VIC 3086, Australia; 4 Department of Anatomical Pathology, NSW Health Pathology, Royal North Shore Hospital, Sydney, NSW 2065, Australia; anthony.james.gill@sydney.edu.au (A.J.G.); angelashihyuan.chou@health.nsw.gov.au (A.C.); 5 Cancer Diagnosis and Pathology Research Group, Kolling Institute of Medical Research, St Leonards, NSW 2064, Australia; 6 Northern Clinical School, Faculty of Medicine and Health, University of Sydney, Sydney, NSW 2050, Australia

**Keywords:** kidney fibrosis, CXCR4, macrophage, i-body, AD-214

## Abstract

Kidney fibrosis is the common pathological pathway in progressive chronic kidney disease (CKD), and current treatments are largely ineffective. The C-X-C chemokine receptor 4 (CXCR4) is crucial to fibrosis development. By using neural cell adhesion molecules as scaffolds with binding loops that mimic the shape of shark antibodies, fully humanized single-domain i-bodies have been developed. The first-generation i-body, AD-114, demonstrated antifibrotic effects in a mouse model of folic acid (FA)-induced renal fibrosis. The second-generation i-body, AD-214, is an Fc-fusion protein with an extended half-life, enhanced activity, and a mutated Fc domain to prevent immune activation. To investigate the renoprotective mechanisms of AD-214, RPTEC/TERT1 cells (a human proximal tubular cell line) were incubated with TGF-b1 with/without AD-214 and the supernatant was collected to measure collagen levels by Western blot. Mice with unilateral ureteral obstruction (UUO) received AD-214 intraperitoneally (i.p.) every two days for 14 days. Kidney fibrosis markers and kidney function were then analyzed. AD-214 suppressed TGF-b1-induced collagen overexpression in RPTEC/TERT1 cells. In UUO mice, AD-214 reduced extracellular matrix (ECM) deposition, restored kidney function, and limited leukocyte infiltration. In a scratch assay, AD-214 also inhibited macrophage migration. To conclude, i-body AD-214 attenuates UUO-induced kidney fibrosis by inhibiting leukocyte infiltration and macrophage migration.

## 1. Introduction

Chemokines and chemokine receptors play crucial roles in immune homeostasis and disease by regulating pro-inflammatory cytokine production and orchestrating the distribution and migration of leukocytes [1,2]. Among chemokine receptors, CXCR4, a member of the G protein-coupled receptors (GPCRs) superfamily, is the most widely expressed [3]. It is expressed on the cell surface of most leukocytes, including all B cells, monocytes, and most T lymphocyte subsets, but only weakly on natural killer (NK) cells [4]. CXCR4 is also found on nonhematopoietic cells such as endothelial cells and epithelial cells, as well as adult stem cells, renal proximal tubular cells, and circulating progenitor epithelial cells [3]. CXCR4 plays a significant role in the pathogenesis of various infectious diseases, cancer, and chronic inflammatory diseases, including CKD [5,6,7,8]. 

CKD is a worldwide public health problem, with adverse outcomes of end-stage kidney disease (ESKD), cardiovascular disease, and premature death [9]. It is estimated that CKD affects 10–14% of the global population, particularly those with diabetes, obesity, or hypertension. Other common causes of CKD include glomerulonephritis (GN) and polycystic kidney disease [9]. Regardless of the initial cause, kidney fibrosis is the hallmark of CKD progression, with ECM accumulation as a major characteristic [10,11]. Collagens are key constituents of ECM, making them important markers that specifically indicate fibrosis in the kidney. These fibrotic changes are strongly correlated with a decline in renal function, making fibrosis a critical predictor of disease progression [11]. Despite many attempts to block kidney fibrosis, few therapeutics have been approved in clinical practice, and a large treatment gap remains [11]. 

CXCR4 is significantly upregulated in fibrotic kidneys and contributes to kidney fibrosis via multiple effectors [6]. Therefore, CXCR4 is regarded as a potential therapeutic target for kidney fibrosis and has received increasing interest from the pharmaceutical industry in developing drugs that specifically target it. Recently, a fully human single-domain antibody-like scaffold termed the “i-body” was developed based on an Ig domain of human neural cell adhesion molecule (NCAM) 1 into which synthetic binding loops that mimic the shark variable new antigen receptor (VNAR) complementarity-determining regions (CDRs). The long CDR3 loop enables the i-body to access groves and cavities of therapeutic targets such as CXCR4, which are typically inaccessible to traditional monoclonal and many other next-generation antibodies [12]. Several CXCR4 antagonistic i-bodies have been reported, which bind deep in the ligand binding pocket of the receptor via the elongated CDR3 loop [12]. 

Animal models are invaluable for exploring potential CKD therapeutic strategies before human trials. Two key models are the FA model and the UUO model. In the FA model, a high dose of FA rapidly induces crystal formation, tubular necrosis, and patchy interstitial fibrosis [13]. The UUO model, characterized by tubular injury caused by obstructed urine flow, also leads to significant renal fibrosis [14]. In rodents, experimental UUO is believed to replicate human chronic obstructive kidney fibrosis, making it a widely used model for studying treatments that reduce fibrosis.

Of interest, the first-generation i-body AD-114 demonstrated an antifibrotic effect and diminished the level of kidney injury in the murine FA-induced renal fibrosis model [15]. An improved i-body, AD-214, has been generated by adding an Fc fragment to AD-114 to extend the half-life and increase manufacturability. Additionally, AD-214 comprises two Fc-linked AD-114 molecules, which enhances its avidity, leading to a higher affinity for CXCR4 and increased activity. Importantly, the Fc domain of AD-214 is designed to be “silent,” preventing antibody-dependent cellular cytotoxicity (ADCC) and antibody-dependent cellular phagocytosis (ADCP), processes that could be harmful in inflammatory and fibrotic diseases. This study aims to investigate further the renoprotective mechanisms of AD-214 in the UUO model to demonstrate that its anti-fibrotic effects are not specific to a single model of kidney injury.

## 2. Results

### 2.1. I-Body AD-214 Mitigates TGF-b1-Induced ECM Accumulation and MCP-1 Synthesis in RPTEC/TERT1 Cells Without Inducing Nephrotoxicity

CXCR4, the specific target of i-body AD-214, plays a central role in the pathogenesis of inflammation and fibrotic diseases [8]. As we reported previously, CXCR4 levels in RPTEC/TERT1 were low in the absence of TGF-b1, whilst exposure to TGF-b1 significantly upregulated CXCR4 expression [15]. Cells were incubated with TGF-b1 with/without AD-214 or AD-114 at a concentration of 1 µM or 2 µM to assess the impact of AD-214 on TGF-b1-induced ECM expression in RPTEC/TERT1. Western blot was conducted to quantify the protein levels of collagen-3 (COL-3) and collagen-4 (COL-4). 

**Figure 1:**
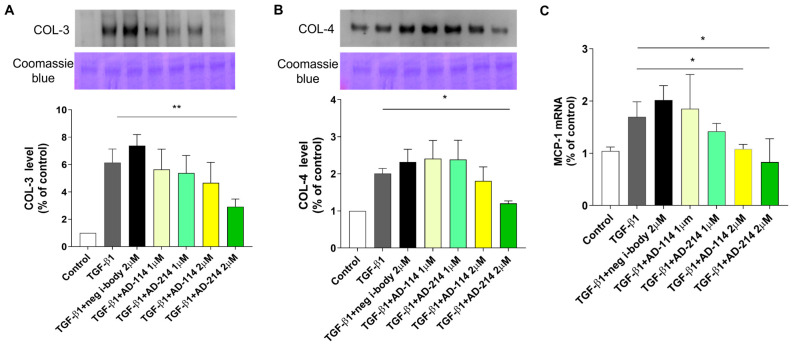
I-body AD-214 inhibits TGF-b1-induced ECM and MCP-1 overexpression. RPTEC/TERT1 cells were exposed to TGF-b1 with AD-114 or AD-214 for 48 h. Supernatants were collected, and COL-3 (**A**) and COL-4 (**B**) were analyzed by Western blot. (**C**) Gene expression of MCP-1 was analyzed by quantitative RT-PCR. The data are presented as mean ± SEM. Statistical analysis was performed using 1-way ANOVA followed by Tukey’s multiple comparisons test. * *p* < 0.05, ** *p* < 0.01, *n* = 4.

As depicted in Figure 1A,B, TGF-b1 stimulation led to a substantial increase in COL-3 and COL-4 secretion. Although the TGF-β1 + neg i-body shows a higher COL-3 and COL-4 level than TGF-β1, there is no statistical significance between the two groups (*p* > 0.05). Compared to TGF-b1, incubation with 2 µM AD-214 significantly reduced COL-3 and COL-4 secretion by 52.4% and 40.1%, respectively (Figure 1A,B, both *p* < 0.05). These findings confirm that TGF-b1 induces fibrotic responses in RPTEC/TERT1 cells, which can be attenuated by concomitant inhibition of CXCR4 with i-body AD-214 at a relatively low concentration of 2 µM (AD-114 has demonstrated anti-fibrotic effects at 3 µM, as previously reported [15]). 

Monocyte chemoattractant protein-1 (MCP-1) is a chemotactic factor for monocytes/macrophages [16], playing a critical role in promoting renal fibrosis by regulating leukocyte migration, proliferation, and differentiation [17]. TGF-b1 has been shown to induce MCP-1 synthesis in proximal tubular cells [18]. Therefore, we evaluated the effects of i-body AD-214 on MCP-1 gene expression in RPTEC/TERT1 cells. As illustrated in Figure 1C, TGF-b1 increased MCP-1 mRNA expression, which was significantly reduced by 51.1% upon treatment with AD-214.

Nephrotoxic biomarkers clusterin, cystatin C (CysC), GSTp, and TIMP-1 can serve as effective in vitro indicators to monitor drug-induced nephrotoxicity in RPTEC/TERT1 cells [19]. To assess whether i-bodies induce nephrotoxicity, the mRNA expression of these markers was measured. As indicated in Supplementary Figure S1, none of these four markers were upregulated by TGF-b1 stimulation or co-incubation with i-body AD-214/AD-114. These results alleviate potential safety concerns regarding i-body AD-214.

### 2.2. CXCR4 Expression Is Upregulated in Mouse Kidneys After UUO 

Experimental UUO in rodents mimics human chronic obstructive nephropathy in an accelerated manner, making it a widely utilized model for investigating treatments that attenuate fibrosis. Following UUO, renal changes, including interstitial inflammation (peak at 2–3 days), tubular dilation, tubular atrophy, and fibrosis, typically occur after seven days. ECM accumulation begins in the interstitial space around seven days post-ligation and continues to increase until 21 days [14]. 

**Figure 2:**
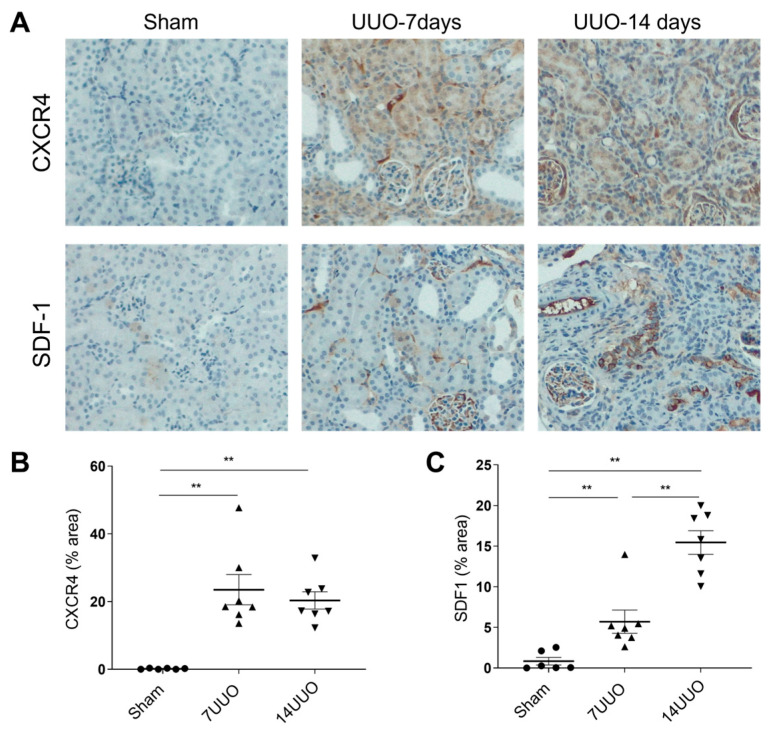
UUO induces significant upregulation of CXCR4 and SDF-1. IHC staining was conducted to measure CXCR4 and SDF-1 expression. (**A**) Representative images of CXC4 and SDF-1 staining in kidneys at days seven and fourteen after UUO. Original magnification: ×200. Quantitation of CXCR4 (**B**) and SDF-1 (**C**) in mouse kidneys at days seven and fourteen after UUO in mice. Statistical analysis was performed using 1-way ANOVA followed by Tukey’s multiple comparisons test. Results are presented as mean ± SEM. ** *p* < 0.01. *n* = 6.

**Figure 3:**
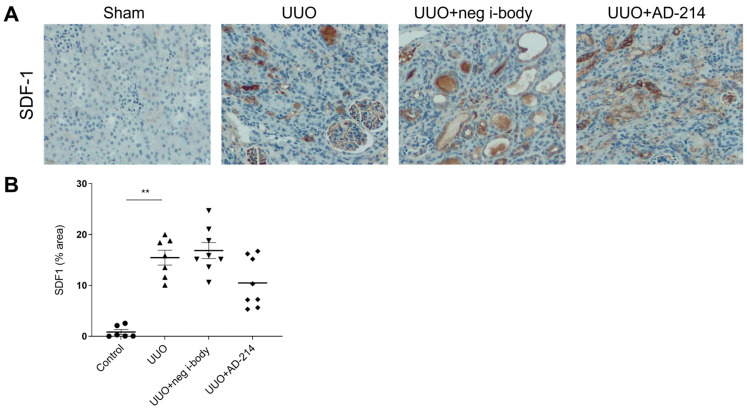
AD-214 does not significantly lower renal SDF-1 expression induced by UUO. Immunohistochemical staining was conducted to measure SDF-1 expression. (**A**) Representative images and (**B**) Quantitation of SDF-1 immunohistochemical staining in kidneys. Original magnification: ×200. Statistical analysis was performed using one-way ANOVA followed by Tukey’s multiple comparisons test. Results are presented as mean ± SEM. ** *p* < 0.01. *n* = 6–8.

In this study, we used the 14-day UUO model. To assess CXCR4 expression after UUO, kidneys from UUO mice were collected at seven and fourteen days post-UUO, and immunohistochemistry (IHC) was conducted accordingly. As illustrated in Figure 2A,B, there was an increase in total kidney CXCR4 expression following UUO. Compared with the sham control, kidneys at day seven and day fourteen UUO exhibited a 23.3-fold and a 20.1-fold increase in CXCR4 expression, predominantly localized to infiltrating immune cells within the interstitium and renal tubular cells. Stromal cell-derived factor-1 (SDF-1, also known as CXCL12) is the principal ligand for CXCR4. As demonstrated in Figure 2A,C, a significant elevation of SDF-1 in kidneys was observed at seven days and fourteen days after UUO (5.8 folds and 17.4 folds increase, respectively). However, this increased level of SDF-1 remained unaffected by the administration of AD-214 (Figure 3, *p* > 0.05), indicating that AD-214 had no impact on the expression of SDF-1.

### 2.3. Blockade of CXCR4 by I-Body AD-214 Ameliorates UUO-Induced Kidney Fibrosis 

The first-generation i-body AD-114 attenuated FA-induced kidney fibrosis when administered at 10 mg/kg daily. Since the i-body alone has a short half-life in blood and lower affinity, we sought to test if the second-generation Fc-fused anti-CXCR4 i-body AD-214 could mitigate renal fibrosis when given at a lower dosage and a longer dosing interval than AD-114. Additionally, several animal models of CKD mimic different clinical manifestations. To confirm the therapeutic potential of treatment and demonstrate that its effect is not model-specific, it is essential to use different models to evaluate the effect and investigate the underlying mechanisms thoroughly. The UUO model is another commonly used mouse model to study anti-fibrosis drugs [14], which we employed in this study. Specifically, from the day following the UUO procedure, mice were dosed i.p. with negative control i-body 21H5-Fc (5 mg/kg) or AD-214 (5 mg/kg) and were then dosed every second day until day fourteen. Specifically, starting the day after the UUO procedure, mice were dosed intraperitoneally with either the negative control i-body 21H5-Fc (5 mg/kg) or AD-214 (5 mg/kg) every second day until day fourteen.

**Figure 4:**
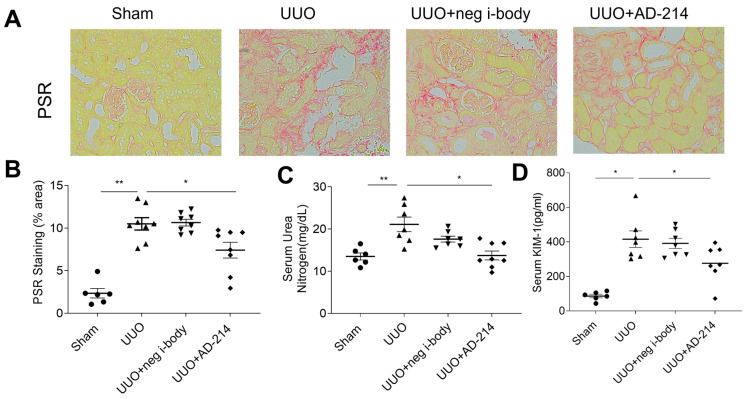
I-body AD-214 ameliorated UUO-induced collagen deposition and improved kidney function. (**A**) PSR staining and (**B**) quantitation. (**C**) Serum BUN. (**D**) Serum KIM-1. BUN: blood urea nitrogen; KIM-1: kidney injury molecule-1. Original magnification: ×200. Statistical analysis was performed using one-way ANOVA followed by Tukey’s multiple comparisons test. Results are presented as mean ± SEM. * *p* < 0.05, ** *p* < 0.01. *n* = 6–8.

Histological assessment is currently the only specific method for fibrosis quantification. Picrosirius red (PSR) staining is a commonly used technique for evaluating renal fibrosis. It is essential for visualizing collagen fibers, making it a key method for assessing collagen buildup in the kidney cortex. As shown in Figure 4A,B, mice treated with AD-214 revealed a marked reduction in collagen deposition (26.2% reduction, *p* < 0.05) relative to the UUO group. As ureter obstruction occurs, blood urea nitrogen (BUN) serves as a surrogate marker of renal function in the UUO model. Blood was collected at the time of sacrifice to assess whether reduced kidney fibrosis correlated with improved physiological parameters, and BUN and kidney injury markers (KIM-1) were measured. UUO mice treated with AD-214 demonstrated a significant reduction in BUN (34.9% reduction) compared to the UUO group (Figure 4C, *p* < 0.05). KIM-1, a type 1 transmembrane protein, undergoes marked, prompt, and specific upregulation in the apical membrane of proximal tubular cells (PTCs) in response to kidney injury and is up-regulated more than any other protein [20]. As indicated in Figure 4D, UUO mice treated with AD-214 exhibited significantly lower levels of KIM-1 (33.5% decrease) compared with UUO mice (*p* < 0.05).

**Figure 5:**
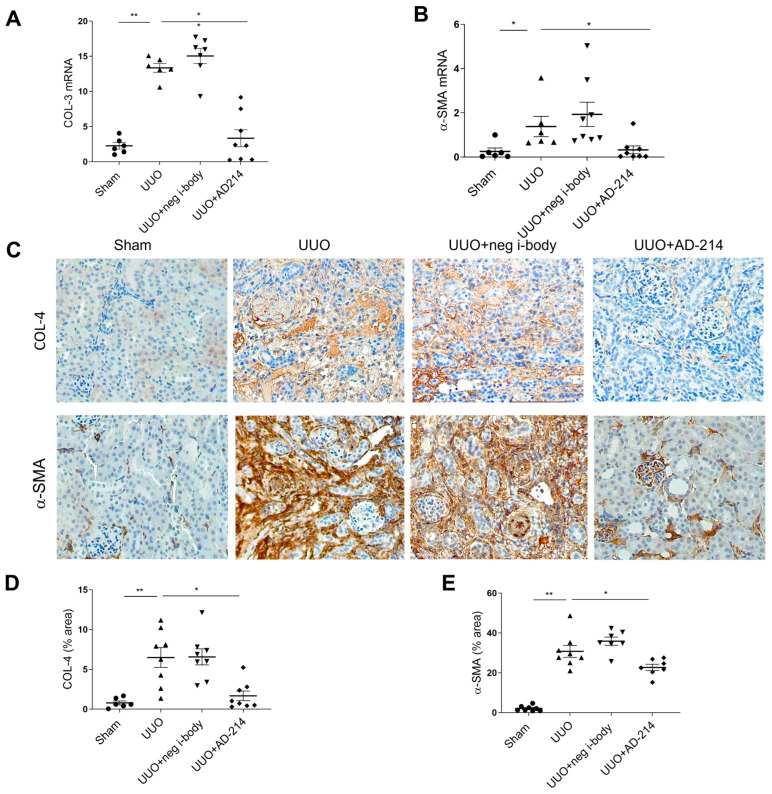
AD-214 attenuates ECM deposition in the UUO model. (**A**,**B**) mRNA expression of COL-3 and a-SMA were measured by quantitative RT-PCR. (**C**) Representative images of COL-4 and a-SMA-stained kidney sections. (**D**,**E**) Quantitation of COL-4 and a-SMA immunohistochemical staining. Original magnification: ×200. Statistical analysis was performed using one-way ANOVA followed by Tukey’s multiple comparisons test. Results are presented as mean ± SEM. * *p* < 0.05, ** *p* < 0.01. *n* = 6–8.

Consistently, smaller increases in COL-3 and a-SMA mRNA were observed in mice administered AD-214 (Figure 5A,B). IHC staining revealed that UUO treatment also induced the deposition of fibrotic markers, including COL-4 and a-SMA. Administration of AD-214 significantly mitigated the COL-4 and a-SMA deposition by 60.2% and 26.3%, respectively (Figure 5C–E, *p* < 0.05), relative to the UUO group. These data suggested that the i-body AD-214 effectively suppressed ECM overproduction and reduced renal fibrosis in the mouse model of UUO.

### 2.4. I-Body AD-214 Attenuates UUO-Induced Kidney Fibrosis by Inhibiting Leukocytes Infiltration

The infiltration of leukocytes expressing CXCR4 plays a pivotal role in mediating tubulointerstitial inflammation and fibrosis in CKD. In the UUO model, the accumulation of leukocytes, especially macrophages and T lymphocytes, in the renal interstitium strongly correlates with the progression of kidney fibrosis [21]. CXCR4, targeted by AD-214, actively participates in the migration and infiltration of these leukocytes into inflamed renal tissue during UUO [6]. To elucidate the impact of i-body AD-214 on the leukocyte infiltration, we conducted IHC to measure the expression of the leukocyte marker CD11b, macrophage marker F4/80, and T lymphocyte marker CD3 in the kidneys. 

**Figure 6:**
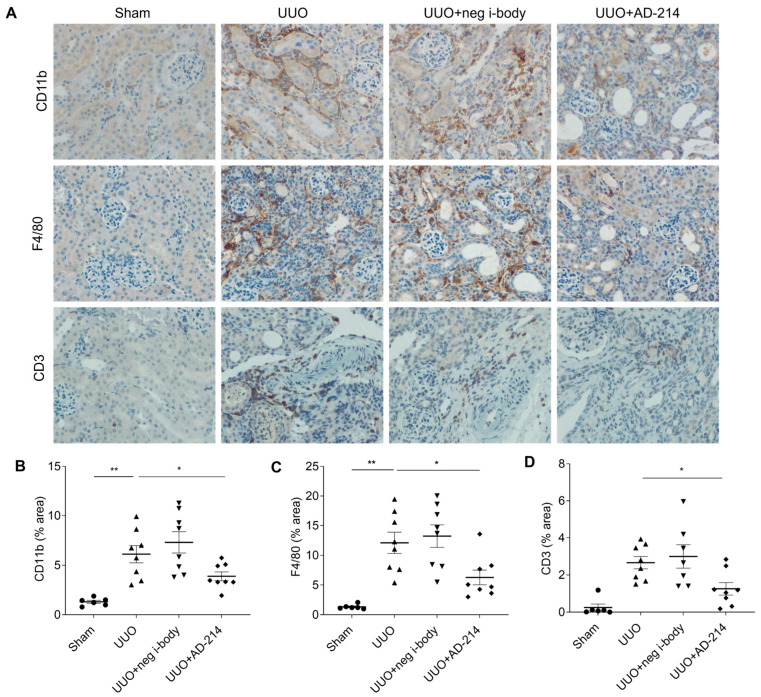
I-body AD-214 ameliorates UUO-induced leukocyte infiltration. (**A**) Representative images of CD11b, F4/80, and CD3-stained kidney sections. Original magnification: ×200. (**B**–**D**) Quantitation. CD11b: leukocyte marker; F4/80: macrophage marker; CD3: T cell marker. Statistical analysis was performed using one-way ANOVA followed by Tukey’s multiple comparisons test. Results are presented as mean ± SEM. * *p* < 0.05, ** *p* < 0.01. *n* = 6–8.

As shown in Figure 6, at day fourteen post-UUO, a prominent infiltration of CD11b-positive leukocytes, F4/80-positive macrophages, and CD3-positive T lymphocytes was observed in the peritubular interstitium in both UUO mice or UUO + negative i-body mice. However, AD-214 treatment led to a significant reduction in the infiltration of leukocytes, macrophages, and T lymphocytes by 31.7%, 44.9%, and 52.8%, respectively, relative to UUO alone. This suggests that the inhibited infiltration of macrophages and T lymphocytes by AD-214 accounts for the main cell types among the various leukocytes.

### 2.5. I-Body AD-214 Inhibited Macrophage Migration 

**Figure 7:**
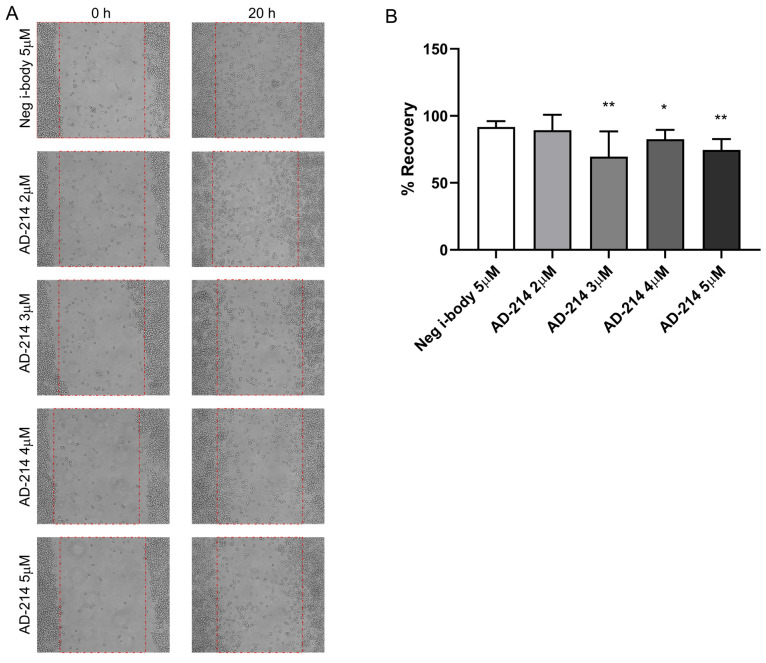
AD-214 inhibits the migration of macrophage cell line RAW264.7 in the scratch assay. (**A**) Scratch areas, marked with red dotted lines, were photographed at 0 h and 20 h after AD-214 treatment. (**B**) The recovery area was quantified using Fiji ImageJ2. Results are presented as mean ± SEM. * *p* < 0.05, ** *p* < 0.01. *n* = 6.

Macrophage migration to inflamed kidneys plays a critical role in promoting kidney fibrosis. A scratch assay was performed using the CXCR4-expressing RAW264.7 macrophage cell line [22,23] to investigate the impact of i-body AD-214 on macrophage migration. A scratch was created at the start, and the scratch area, marked with red dotted lines (Figure 7A), was captured and analyzed at 0 and 20 h. As depicted in Figure 7B, the results showed that the area covered by cells (recovery percentage) in the AD-214 treatment groups (3 μM, 4 μM, and 5 μM) was 69.6%, 82.6%, and 74.6%, respectively. These were significantly lower than the negative i-body control group (91.7%, all *p* < 0.05). These findings indicated that the suppression of macrophage migration is one of the mechanisms by which AD-214 reduced leukocyte infiltration.

## 3. Discussion

GPCRs represent the largest and most diverse group of receptors identified in humans. They are intensively studied as drug targets, primarily due to their substantial role in human pathophysiology and their pharmacological accessibility [24]. Recent advances in structural biology and advancements in biotechnology have paved the way for novel avenues in GPCR drug discovery. The emergence of next-generation antibody-based modalities, including protein scaffolds, bispecific antibodies, antibody-drug conjugates (ADCs), and chimeric antigen receptor T-cell therapy (CAR-T), have emerged as promising targeting strategies [25].

The GPCR CXCR4 is a promising therapeutic target for kidney fibrosis [6]. CXCR4 is appreciably upregulated in multiple models of kidney fibrosis [6,15]. I-bodies are a novel class of humanized next-generation antibody-like proteins with the potential to overcome some of the limitations of monoclonal antibodies in therapeutics [12]. The anti-CXCR4 i-body AD-114 was developed and demonstrated to have anti-fibrotic activity in a mouse model of pulmonary fibrosis, and in one of our previous studies [26], we demonstrated the anti-fibrotic effect of i-body AD-114 in an FA-induced CKD model [15]. AD-214, an Fc-fusion protein comprising a dimer of two AD-114 i-body molecules, exhibits an extended half-life and enhanced activity. 

Testing the therapeutic efficacy of the i-body in more than one model demonstrates that the effect is not model-specific and establishes the generalizability of this approach to multiple forms of kidney fibrosis. Hence, in this study, we evaluated the anti-fibrotic effect of the i-body AD-214 in vitro RPTEC/TERT1 cell lines and an in vivo obstructive model-UUO. To determine the optimal concentration of AD-214, we conducted several preliminary studies. At the concentration of 2 µM or lower, the i-body AD-114 did not inhibit TGF-b1-induced ECM upregulation. However, co-incubation with AD-114 at 3–5 µM significantly inhibited TGF-b1-induced ECM compared to the negative control i-body [15]. Since AD-214 has enhanced activity, we hypothesized it would be effective at lower concentrations than AD-114. To test this, we investigated its effect at the concentrations of 2 µM and 1 µM. A similar rationale applied to animal studies. In a previous publication, AD-114 attenuated FA-induced kidney fibrosis at a dose of 10 m/kg [15]. For the animal study with AD-214, we adjust the dosage, testing 5 mg/kg and 1 mg/kg. At 1 mg/kg, AD-214 showed no effect. 

Primary RPTEC cells undergo replicative senescence in culture. The RPTEC/TERT1 cell line is derived from immortalized renal proximal tubule epithelial cells (RPTEC) of a healthy human donor using the catalytic subunit of human telomerase reverse transcriptase (hTERT), which is responsible for telomere stabilization [27]. This cell line exhibits functional similarity to native proximal tubule cells, including prototypical RPTEC structural and biochemical properties [27]. In this study, we used the RPTEC/TERT1 cell line to evaluate the impact of i-body in vitro. Previously, we reported that i-body AD-114 reduced the TGF-b1-induced secretion of fibrotic markers at a concentration of 3 µM [15]. Our findings in this study indicate that AD-214 effectively mitigates the fibrotic response by downregulating the profibrotic factor TGF-b1-induced fibrotic markers COL-3 or COL-4 at a lower concentration of 2 µM, suggesting enhanced functionality as anticipated. Implementing an in vitro assay predictive of nephrotoxicity in the early drug discovery stages of drug discovery could potentially reduce attrition rates in later stages of drug development. In this study, we examined four nephrotoxicity markers-clusterin, CysC, GSTp, and TIMP-1-in RPTEC/TERT1 cell culture treated with i-body AD-214. Our findings revealed no nephrotoxicity, promoting us to proceed with in vivo animal studies with i-body AD-214.

Renal fibrosis is the common pathway underlying the progression of various CKD. In this study, a UUO mouse model was employed to cause renal fibrosis, where one side of urine flow is obstructed to trigger primary tubular injury and kidney fibrosis at a later stage. Following UUO, we observed an upregulation of CXCR4 in fibrotic kidneys. Subsequent administration of anti-CXCR4 i-body AD-214 effectively mitigated kidney fibrosis, as evidenced by diminished collagen staining, decreased ECM protein expression, and attenuation of UUO-mediated elevation in BUN and KIM-1 levels.

The progression of kidney fibrosis is typically accompanied by interstitial leukocytic cell infiltrates, which exacerbate the condition by the production of proinflammatory, proapoptotic, and profibrotic mediators [28]. The recruitment of leukocytes into the kidney involves local expression of chemokines, which interact with corresponding chemokine receptors on the leukocyte’s surface. As chemokine receptors can bind to multiple proinflammatory chemokines, therapeutic intervention targeting chemokine receptors rather than individual chemokine ligands may be a more effective strategy to disrupt leukocyte recruitment to sites of tissue injury. CXCR4, a chemokine receptor expressed on most leukocytes, plays a crucial role in orchestrating the trafficking of leukocytes to sites of injury [29]. For example, CXCR4 is important in the migration of neutrophils from within the lung tissue across the epithelium into the alveolar spaces [30]. Another study in mice showed that a subpopulation of CXCR4hi neutrophils constituted the first line of defense by rapid migration to the site of inflammation during an acute inflammatory response [31]. A recent study indicated that CXCR4 signaling is a critical checkpoint for Langerhans cell and dendritic cell (DC) migration from the skin to lymph nodes [32]. Moreover, CXCR4 is involved in the inappropriate retention of activated innate inflammatory cells, such as neutrophils, at inflammation sites [33]. Hence, CXCR4 has been a target of interest in inflammation. CD11b belongs to the integrin family and is expressed on most leukocytes, including neutrophils, monocytes, NK cells, and a subset of lymphocytes [34]. Among infiltrating leukocytes, macrophages and lymphocytes comprise the major populations of immune infiltrates [14]. In this study, we demonstrated that UUO mice administrated AD-214 had a significant reduction in leukocyte, T lymphocytes, and macrophage infiltration, as evidenced by decreased CD11b, CD3, and F4/80 expression. This is consistent with previous studies that suggest CXCR4 is involved in the trafficking of leukocytes to injured sites [15].

Cell migration is a crucial process essential for cells to navigate and reach their appropriate location within a given environment to fulfill their functions [35]. Macrophage migration toward inflamed tissues is crucial for rapid phagocytosis of pathogens and apoptotic materials, contributing to protective immunity. However, in pathological conditions, macrophage movement can also have adverse effects, such as tissue damage in chronic inflammation [36]. Ongoing kidney damage can cause continuing macrophage infiltration in a vicious cycle that leads to the destruction of the normal kidney tissue structure and irreversible tissue fibrosis. Hence, in conditions like kidney fibrosis, macrophage migration to the inflamed kidney plays a critical role in promoting disease progression [36]. The macrophage cell line RAW264.7 expresses CXCR4. In this study, we conducted a scratch assay to evaluate the impact of i-body AD-214 on macrophage migration. Our findings reveal that 3–5 µM AD-214 inhibited the scratch-induced migration of RAW264.7, indicating that inhibiting macrophage migration is one of the mechanisms by which AD-214 reduces leukocyte infiltration. However, there is no strong correlation between the concentration of AD-214 applied and the percentage of cell recovery, which could be due to several factors. First, the saturation effect may occur at higher concentrations, where the binding sites for AD-214 on target cells become saturated, leading to a plateau in effectiveness. Beyond this point, increasing the concentration may not significantly enhance cell recovery. Additionally, the relationship between AD-214 concentration and its effect might not be linear. Factors such as CXCR4 receptor internalization or downstream signaling saturation could lead to diminishing returns at higher concentrations.

The NLRP3 inflammasomes are a complex composed of NLRP3, caspase-1, and adapter apoptosis-associated speck-like protein (ASC) that plays a role in linking NLRP3 and caspase-1. It can respond to the stimulation of damage-associated molecular patterns (DAMP) or pathogen-associated molecular patterns (PAMPs) during infection or tissue damage [37]. The activation of the NLRP3 inflammasome leads to pyroptosis and the release of inflammatory factors such as IL-1β and IL-18, which are involved in the inflammatory response [37]. Growing evidence has revealed that the NLRP3 inflammasome significantly contributes to the progression of kidney fibrosis [38,39,40]. The immunoprecipitation assay indicated a direct interaction between CXCR4 and TXNIP [41]. Recent studies have increasingly implicated CXCR4 in the modulation of the NLRP3 inflammasome. CXCR4 siRNA and CXCR4 antagonist-AMD3100 effectively downregulated the NLRP3 pathway in kidney injury induced by BDE-47 [42]. In myocardial infarction inflammation, CXCR4 upregulation activates the nuclear translocation and phosphorylation of NF-κB, thus promoting NLRP3 inflammasome activation [43]. In addition, CXCR4 was found to promote TXNIP expression, thereby activating the NLRP3 inflammasome. CXCR4 blockade by loganin suppressed CXCR4-dependent TXNIP-induced NLRP3 inflammasome activation [44]. These findings are consistent with a previous study where CXCR4 knockdown inhibited the upregulation of TXNIP and NLRP3 inflammasome activation [41]. Thus, in addition to investigating the inhibition of leukocyte infiltration, we also explored the impact of AD-214 on the activation of NLRP3 inflammasomes. Although NLRP3 expression increased in UUO mice, administration of AD-214 did not alter the expression of NLRP3, ASC, or caspase-3, indicating that AD-214 has no effect on NLRP3 inflammasome activation. 

Furthermore, CXCR4 induces epithelial-mesenchymal transition (EMT) through activation of the Wnt/β-catenin signaling pathway in rat chronic allograft nephropathy [45], consistent with another report suggesting CXCR4 involvement in the JAK/STAT/GSK3β/β-catenin pathway activation in the UUO model [46]. Investigation into the involvement of AD-214 in these pathways is ongoing.

To conclude, our findings demonstrate that i-body AD-214 attenuates UUO-induced kidney fibrosis by inhibiting leukocyte infiltration and macrophage migration. Further research into the mechanisms of AD-214 using additional animal models, such as diabetic kidney models and human kidney organoids to mimic the human kidney environment, is warranted to deepen our understanding of the anti-fibrotic and anti-inflammatory mechanisms of AD-214 and to identify biomarkers useful in clinical trials for renal fibrosis. However, when taken together, the results herein and those from the recently completed Phase 1 clinical trials for AD-214 in healthy volunteers, which demonstrate an excellent safety and pharmacokinetic/pharmacodynamic (PK/PD) profile, support the progression of AD-214 to Phase 2 trials in patients with renal fibrosis. 

## 4. Materials and Methods

### 4.1. I-Body 

AD-214, AD-114, and a nonspecific negative control i-body 21H5-Fc (21H5 was fused at its C-terminus with a human IgG1 mutant Fc region) were supplied by AdAlta Limited (Melbourne, Australia).

### 4.2. Cell Culture 

RPTEC/TERT1 cell lines (ATCC^®^ CRL4031™), a human proximal tubular cell line, ectopically express the catalytic subunit of telomerase (TERT). RAW264.7 cell line is a mouse macrophage cell line established from a tumor in a male mouse induced with the Abelson murine leukemia virus. These two cell lines were cultured according to the instructions from ATCC in a humidified atmosphere with 5% CO_2_.

### 4.3. In Vitro Model of Kidney Fibrosis 

RPTEC/TERT1 cells were seeded into six well-plates and incubated with/without recombinant human TGF-b1 (2 ng/mL) in the presence or absence of i-bodies AD-214, negative control i-body 21H5-Fc or AD-114 (for comparison) for 48 h. Then, the culture supernatants, total RNA, and cell lysates were collected, respectively.

### 4.4. Animal Experiments 

**Figure 8:**
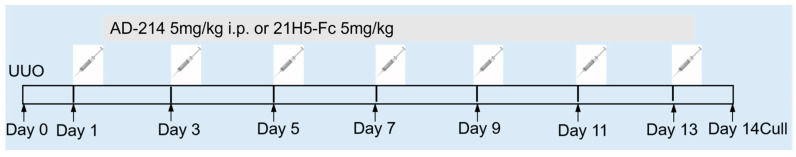
Flow charts of animal experimental design. UUO was induced at day 0. From the following day of UUO (day 1), mice were dosed i.p. once with either AD-214 or negative control i-body 21H5-Fc once every two days until day fourteen.

Male C57BL/6 mice, 6–8 weeks old, weighing 20–25 g, were randomized by body weight and divided into treatment groups (*n* = 6–8 per group). To evaluate the effects of i-body AD-214 on renal fibrosis, unilateral ureteral obstruction (UUO) was induced at day 0 according to published methods. The left ureter was isolated and tied off 0.5 cm from the pelvis, while the right ureter was left unclamped and served as the sham-operated control. The day following induction of UUO, mice were dosed i.p. once either with negative control i-body 21H5-Fc (5 mg/kg) or AD-214 (5 mg/kg) every two days until day 14 (Figure 8). Kidneys were then harvested and fixed by immersion in 10% phosphate-buffered formalin for further analyses.

### 4.5. Biomarker Assays of Kidney Function and Damage

The BUN was measured by the BUN Colorimetric Detection Kit (Thermo Fisher Scientific, Waltham, MA, USA), and the plasma KIM-1 was measured by ELISA (Abcam, Cambridge, UK), as per the manufacturers’ instructions. 

### 4.6. RNA Isolation and Real-Time PCR (RT-PCR) Analysis 

Total RNA was extracted from cells or mouse kidney cortex tissues using the RNeasy mini kit (Qiagen, Germantown, MD, USA). cDNA was synthesized with an iScript cDNA synthesis kit (Bio-rad, Hercules, CA, USA). Quantitative RT-PCR was performed using the SYBR Green Master Mix (Bio-rad) with the intron-spanning primers, as shown in Supplemental Table S1 on the ABI-Prism-7900 Sequence Detection System (Applied Biosystems, Waltham, MA, USA). The relative mRNA expression level was calculated using QuantStudio™ 12K Flex Software. Glyceraldehyde 3-phosphate dehydrogenase (GAPDH) and b-Actin were used as endogenous control genes for human RPTEC/TERT1 cells and mice, respectively.

### 4.7. Western Blot Analysis

COL-3 and COL-4 were measured in cell culture supernatant. Samples were separated by SDS-PAGE and then transferred to the Hybond ECL nitrocellulose membrane (Amersham, UK). The membranes were incubated with primary antibodies against COL-3 (Abcam, ab7778), COL-4 (Abcam, Cambridge, UK, ab6586), and a-Tubulin (Abcam, ab56676) at 4 °C overnight, followed by 1 h of incubation with HRP-conjugated secondary antibody at room temperature. The blots were then visualized using standard enhanced chemiluminescence (ECL) methodology; a-Tubulin protein was used as the endogenous control.

### 4.8. PSR Staining and IHC

Kidneys were fixed in 4% paraformaldehyde and embedded with paraffin. About 4 μm kidney sections were cut and stained as follows. After deparaffinization with xylene, slides were immersed in decreasing concentrations of ethanol (100%, 100%, 95%, and 70%) and rinsed in a container of running water. PSR staining (24901, Polysciences, Warrington, PA, USA) was undertaken according to the manufacturer’s protocol. For IHC, kidney slides were then incubated in citrate buffer (PH 6, heated to 99 °C) for epitope retrieval and 0.3% hydrogen peroxide to block endogenous peroxidase activity. After preincubation with 10% protein block (Dako) to block nonspecific binding of antibodies, the tissues were incubated overnight at 4 °C with primary antibodies against CXCR4 (Santa Cruz, Dallas, TX, USA, sc-53534), SDF-1 (Santa Cruz, sc-74271), a-SMA (Sigma, St. Louis, MO, USA, A2547), COL-4 (Abcam, ab6586), CD11b (Abcam, ab133357), F4/80 (Abcam, ab111101), CD3 (Abcam, ab16669). Slides were then washed and incubated with secondary antibodies EnVision + System-HRP Labelled Polymer Anti-rabbit (Dako, Santa Clara, CA, USA, K4003) or EnVision + System-HRP Labelled Polymer Anti-mouse (Dako, K4001). After washing with TBST, kidney sections were covered with the DAB (Dako) for 10 min to produce a brown color. Ten randomly chosen fields of kidney cortex were captured per mouse, and staining was quantified as the percentage of the total area using Fiji ImageJ.

### 4.9. Scratch Assay

The experiments were performed as described in [47]. Briefly, RAW264.7 cells (3 × 10^5^ cells/well) were seeded in a 6-well culture plate (Corning-Costar, Corning, NY, USA) for 24 h to reach 80% confluences. Scratched wound lines were created using a 200 μL micropipette tip. Then, cells treated with i-body AD-214 or negative control i-body 21H5-Fc were cultured for 20 h. The wounded area was visualized using a Nikon Eclipse TS2-LS microscope equipped with NIS-Elements 3.0 software (Tokyo, Japan) and was calculated using Fiji ImageJ software. Cell motility was estimated by the quantification of the % of recovery using the equation: R (%) = [1 − (wound area at Tt/wound area at T0)] × 100, where T0 is the wounded area at 0 h, and Tt is the wounded area after 20 h.

### 4.10. Statistics

Column graphs and plots were created using GraphPad Prism 10. Data are shown as mean ± SEM. Statistical analysis of data from two groups was compared using the Student’s unpaired 2-tailed *t*-test. Data from multiple groups were analyzed by one-way ANOVA, followed by Tukey’s multiple comparisons test. Statistical significance was determined as *p* < 0.05.

### 4.11. Study Approval

All animal experiments were performed in accordance with the National Health and Medical Research Council of Australia’s Code for the Care and Use of Animals for Scientific Purposes and were approved by the Northern Sydney Local Health District Animal Ethics Committee (NSLHD AEC, NSLHD reference: RESP/16/300).

## Data Availability

The data that support the findings will be available in pubmed following an embargo from the date of publication to allow for commercialization of research findings.

## Supplementary Materials

The following supporting information can be downloaded at: https://www.mdpi.com/article/10.3390/ijms252313127/s1.
