# Supplementary material for: Second Generation I-Body AD-214 Attenuates Unilateral Ureteral Obstruction (UUO)-Induced Kidney Fibrosis Through Inhibiting Leukocyte Infiltration and Macrophage Migration
Source: Int J Mol Sci. 2024 Dec 6;25(23):13127. doi: 10.3390/ijms252313127 (PMC11641963; doi:10.3390/ijms252313127)
Supplement: Supplementary file 1 [file ijms-25-13127-s001.zip › ijms-3327806-supplementary.pdf]

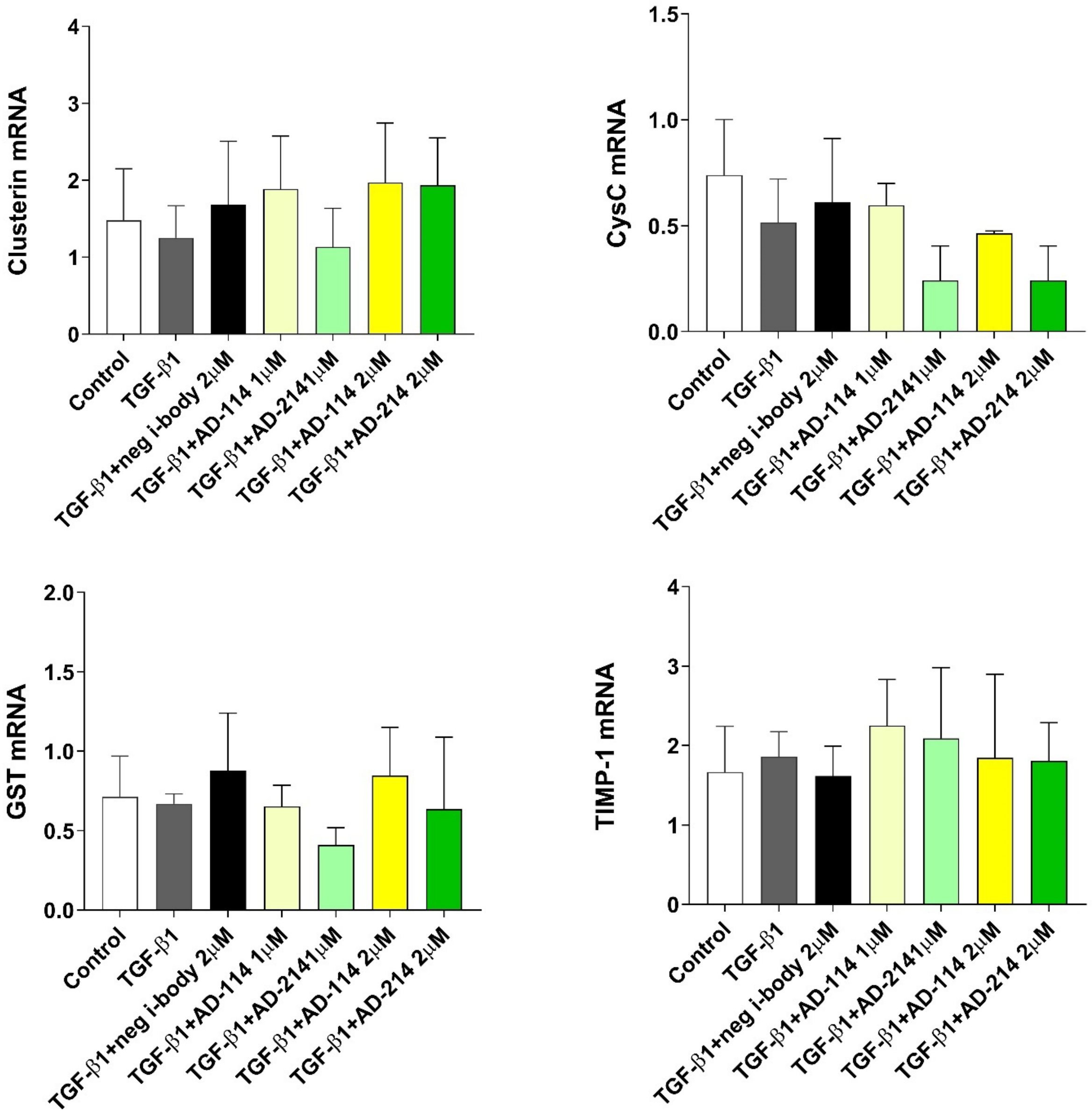

Figure S1. The mRNA expression levels of clusterin, CysC, GSTπ and TIMP-1 were measured in RPTEC/TERT1 cell treated with TGF-β1 with/without i-bodies. Results are presented as mean±SEM. n = 4.

Table S1. The sequence of primers used for quantitative RT-PCR.

Species	Target	Forward (5'-3')	Reverse (5'-3')
**Human**	**GAPDH**	AGCCACATCGCTCAGACAC	GCCCAATACGACCAAATCC
	**TIMP-1**	GGGGCTTCACCAAGACCTAC	GGAAGCCCTTTTCAGAGCCT
	**CysC**	GCCTGTGCCTATCACCTCTTAT	CCTTCTCTGTCTGTCTCCTGGT
	**Clusterin**	CCAGGACAGGTTCTTCACCC	CGTACGGAGAGAAGGGCATC
	**GST-π**	TATTTCCCAGTTCGAGGCCG	TACAGGGTGAGGTCTCCGTC
**Mouse**	**β-Actin**	CAGCTGAGAGGGAAATCGTG	CGTTGCCAATAGTGATGACC
	**COL-3**	TCCCCTGGAATCTGTGAATC	TGAGTCGAATTGGGGAGAAT
	**α-SMA**	CATCTTTCATTGGGATGGAG	TTAGCATAGAGATCCTTCCTG
